# Recent Progress on Chemical Production From Non-food Renewable Feedstocks Using *Corynebacterium glutamicum*

**DOI:** 10.3389/fbioe.2020.606047

**Published:** 2020-12-10

**Authors:** Bin Zhang, Yan Jiang, Zhimin Li, Fei Wang, Xiao-Yu Wu

**Affiliations:** ^1^College of Bioscience and Bioengineering, Jiangxi Agricultural University, Nanchang, China; ^2^Jiangxi Engineering Laboratory for the Development and Utilization of Agricultural Microbial Resources, Jiangxi Agricultural University, Nanchang, China

**Keywords:** *Corynebacterium glutamicum*, renewable feedstocks, metabolic engineering, chemical, fermentation

## Abstract

Due to the non-renewable nature of fossil fuels, microbial fermentation is considered a sustainable approach for chemical production using glucose, xylose, menthol, and other complex carbon sources represented by lignocellulosic biomass. Among these, xylose, methanol, arabinose, glycerol, and other alternative feedstocks have been identified as superior non-food sustainable carbon substrates that can be effectively developed for microbe-based bioproduction. *Corynebacterium glutamicum* is a model gram-positive bacterium that has been extensively engineered to produce amino acids and other chemicals. Recently, in order to reduce production costs and avoid competition for human food, *C. glutamicum* has also been engineered to broaden its substrate spectrum. Strengthening endogenous metabolic pathways or assembling heterologous ones enables *C. glutamicum* to rapidly catabolize a multitude of carbon sources. This review summarizes recent progress in metabolic engineering of *C. glutamicum* toward a broad substrate spectrum and diverse chemical production. In particularly, utilization of lignocellulosic biomass-derived complex hybrid carbon source represents the futural direction for non-food renewable feedstocks was discussed.

## Introduction

**FIGURE 1:**
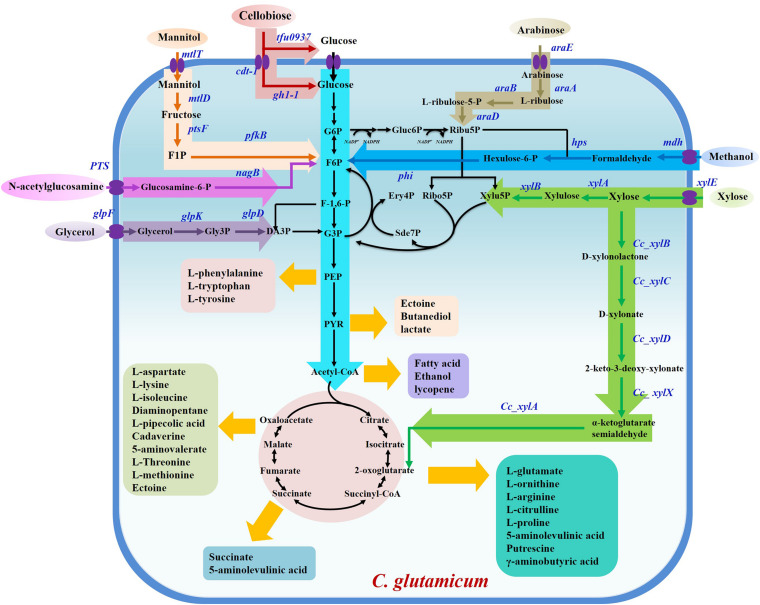
Overview of non-food renewable carbon resource utilization and chemicals production in *C. glutamicum*. G6P, glucose-6-phosphate; F6P, fructose-6-phosphate; F-1,6-P, fructose-1,6-diphosphate; G3P, glyceraldehyde-3-phosphate; DA3P, dihydroxyacetone-3-phosphate; PEP, phosphoenolpyruvate; PYR, pyruvate; Gluc6P, gluconate-6-phosphate; Ribu5P, L-ribulose-5-phosphate; Ribo5P, ribose-5-phosphate; Xylu5P, xylulose-5-phosphate; Ery4P, erythritol-4-phosphate; Sde7P, sedoheptulose-7-phosphate; F1P, fructose-1-phosphate; Gly3P, glycerol-3-phosphate.

Since isolation in 1957, *Corynebacterium glutamicum* has been intensively applied for biobased chemical production due to its ability to secrete amino acids, which are traditionally used as drugs or health products (Kogure and Inui, 2018; Wu et al., 2019). These amino acids and their derived chemicals are worth billions of dollars per year. Development of engineered strains with higher production performance is an active field that has attracted numerous researchers (Lee and Wendisch, 2017b; Li et al., 2017; D’Este et al., 2018; Zhang et al., 2018a,b). In particular, most amino acids, including L-glutamate, L-lysine, L-arginine, L-valine, and L-ornithine, have achieved industrial-scale production due to rapid development of gene-editing and fermentation-manipulation techniques. These amino acids are used in human nutrition, food additives, and drug preparation, applications that benefit from the use of biologically safe *C. glutamicum* (Lee and Wendisch, 2017a). In addition to amino acids, *C. glutamicum* has also been extensively modulated to produce a multitude of valuable products, including bulk chemicals, natural products, polymers, proteins, and biofuels (Becker et al., 2018b; Shanmugam et al., 2018, 2019; Wendisch et al., 2018). This vigorous development has benefited from fossil fuel depletion and anthropogenic climate change caused by the emission of toxic gases generated from oil decomposition, which traditionally served as the major source of manufactured chemicals (Sun et al., 2018). However, biobased production of metabolites using *C. glutamicum* consumes large amounts of glucose, obtained from the hydrolysis reaction of starch, creating competition for food with humans. Hence, it is critical to exploit alternative renewable carbon sources, such as agricultural wastes, industrial wastes, and others for the cultivation of industrial model strains. In the past few years, research efforts have shifted toward biobased production of metabolites from non-food renewable feedstocks. Here are summarized recent advances in the utilization of alternative C-resources, including xylose, arabinose, methanol, glycerol, and mannitol, to produce high-value chemicals using *C. glutamicum* (Figure 1).

## Bioconversion of Single-Carbon Sources to Valuable Chemicals

### Xylose

Xylose, a pentose, is the major constituent of lignocellulose biomass, and was deemed to be the second most abundant biological resource, after glucose (Elmore et al., 2020). High-value utilization of xylose is shifting from bioenergy to a broader range of chemicals. Engineering microbes to produce biobased chemicals from xylose provides a potential alternative to threatening human food security by continuing to use glucose. In *C. glutamicum*, the absence of xylose isomerase means there is no established endogenous metabolic pathway for xylose assimilation. To overcome this obstacle, *xylA*, which encodes xylose isomerase and originates from various microorganisms, has been amplified and employed to reconstruct the xylose utilization metabolic pathway in *C. glutamicum* (Zhao et al., 2018). Heterologous expression of *xylA* from *Xanthomonas campestris* converts imported xylose into xylulose, demonstrating better performance than *xylA* from other microbial species. Tandem expression of *X. campestris xylAB* operon by employing a plasmid or chromosomal insertion of gene copy approach enables impressive conversion of xylose to chemicals in *C. glutamicum* (Table 1). For instance, we previously demonstrated plasmid-based expression of *X. campestris xylAB* operon in the L-ornithine-producing strain *C. glutamicum* SO26 that significantly improved the yield of L-ornithine. By adjusting the expression of the *xylAB* operon, we achieved an L-ornithine titer of 18.9 g/L, representing the highest L-ornithine production titer from xylose recorded to date (Zhang et al., 2019). In contrast to the xylose isomerase (XI) pathway, the Weimberg (WMB) pathway, consisting of multistep reactions catalyzed by enzymes including xylose dehydrogenase, xylonolactonase, xylonate dehydratase, 2-keto-3-deoxy-D-xylonate dehydratase, and α-ketoglutarate semialdehyde dehydrogenase, was introduced into *C. glutamicum* to progressively convert xylose to α-ketoglutarate (Figure 1; Zhao et al., 2018; Choi et al., 2019). The enzymes involved in the WMB pathway are encoded by genes listed as belonging to the *xylXABCD* operon in *Caulobacter crescentus*. Heterologous expression of the *xylXABCD* operon allowed *C. glutamicum* to utilize xylose without loss of carbon; however, cell growth was hindered by inferior metabolic flux. To overcome this growth inhibition, the mutated strain *C. glutamicum* WMB2evo was selected by adaptive laboratory evolution, providing a 3.7-fold increase in growth rate compared to the original strain (Radek et al., 2017). Results from comparative genomics analysis suggest that mutations in IolR, which upregulated the expression of *iolT1*, significantly improved xylose transport and contributed to increased cell growth (Radek et al., 2017). It is universally acknowledged that reinforcing the xylose uptake system markedly increases conversion from xylose to valuable chemicals. Currently, two transmembrane proteins capable of transporting xylose, XylE (Yim et al., 2016) and AraE (Chen Z. et al., 2017), are applied to boost xylose utilization, which promotes the accumulation of metabolites. *C. glutamicum* ATCC 31831, possessing the endogenic arabinose transporter AraE, and *C. glutamicum* ATCC 13032, which lacks this transporter, were simultaneously modulated to produce xylonic acid from xylose; as a result, engineered *C. glutamicum* ATCC 31831 produced approximately 10% more xylonic acid than engineered *C. glutamicum* ATCC 13032 (Sundar et al., 2020). During 120 h of fermentation, 75% xylose consumption in the engineered *C. glutamicum* ATCC 31831 strain likewise outstripped the 60% xylose consumption observed in the AraE-absent *C. glutamicum* ATCC 13032 strain. It can be widely accepted that *C. glutamicum* ATCC 31831 is a superior host for the conversion from xylose to valued chemicals. A robust uptake system exerts the crucial role in xylose utilization by using *C. glutamicum*. Although xylose is the second abundant carbon source next to glucose, the fermentation of pure xylose to produce compounds has been little investigated. This probably due to xylose generally derives from complex carbon sources, frequently obtained by the pretreatment of cellulose, rather than pure sugars like glucose.

**TABLE 1:** Directly conversion from xylose to chemicals by using *C. glutamicum*.

**Strains (*C. glutamicum*)**	**Products**	**Modulations**	**Titer (g/L)**	**Yield (g/g)**	**Cultivation**	**References**
ATCC 31831 pVWEx1-*xylB*	Xylonic acid	Overexpression of *xylB*	56.32	0.94	Shake flask; Batch	Sundar et al., 2020
SL-1A pIEARKT	Ethylene Glycol	Heterologous overexpression of D-tagatose 3-epimerase, L-fuculose kinase, and YqhD reductase	5.80	0.31	Shake flask; Batch	Lee et al., 2019
SL-3 pIEAKD pZ8-AY	Glycolate	Heterologous overexpression of D-tagatose 3-epimerase, L-fuculose kinase	19.20	0.96	BioLector; Batch	Lee et al., 2020
SO29	L-ornithine	Heterologous overexpression of *xylAB* operon	18.90	0.40	Shake flask; Batch	Zhang et al., 2019
SAR3	Sarcosine	Heterologous expression of *dpkA* and overexpression of *xylAB* operon	8.7	0.25*	Shake flask; Batch	Mindt et al., 2019b
HalT1 (pECXT99A-*xylAB*)	Halogenated tryptophan	Heterologous expression of *rebH* and *rebF*; overexpression of *xylAB* operon	0.034	ND	Shake flask; Batch	Veldmann et al., 2019
BETALYS	Carotenoids	Deletion of *crtR*, *cg0719*, *cg0718*, and *cg0717*	0.007	0.0007*	Shake flask; Batch	Henke et al., 2018
Ecto5	Ectoine	Overexpression of *ectABC* operon and overexpression of *xylAB* operon	0.4	ND	Shake flask; Batch	Perez-Garcia et al., 2017
BL-1 pXylAB	Succinate	Deletions of the *pqo*, *pta-ack*, *sdhCAB*, and *cat*; overexpression of *xylAB* operon	7.22	0.18	Shake flask; Batch	Jo et al., 2017
MH15	3-Hydroxypropionic acid	Overexpression of AraE and *xylAB* operon	35.4	0.44	Shake flask; Batch	Chen Z. et al., 2017
Cg-xr3	Xylitol	Introduction of pentose transport and xylitol synthesis pathway	6.2	0.96	Shake flask; Batch	Dhar et al., 2016
DAP-Xyl2	1,5-Diaminopentane	Overexpression of *tkt* and *fbp*; attenuation of *icd*; deletion of *act* and *lysE*	103	0.55	Bioreactor; Fed-batch	Buschke et al., 2013
PUT21	Putrescine	Overexpression of *xylA* and *xylB* from different host	3.78	ND	Shake flask; Batch	Meiswinkel et al., 2013a

*These values were not described in the main text of the original reference and thus estimated from the figure or graph.

### Methanol

Methanol is an attractive alternative feedstock that can be easily obtained from the oxidation of methane. Ongoing development of natural gas, the main source of methane, and use of exploitation technology enable the steady conversion from methane to methanol, continuously reducing the price of methanol (Zhao et al., 2018). Consequently, microbes engineered to convert methanol to valuable metabolites have received considerable attention regarding their comparable cost to glucose-based processes. In microbes, the ribulose monophosphate (RuMP) pathway demonstrates advantages in generating ATP and NAD(P)H, which are frequently used for methanol assimilation, a process in which methanol is converted to fructose 6-phosphate, catalyzed by methanol dehydrogenase (encoded by *mdh*), 3-hexulose-6-phosphate synthase (encoded by *hps*), and 6-phospho-3-hexuloisomerase (encoded by *phi*) (Figure 1; Zhao et al., 2018). In addition to natural occurrence in methylotrophic microorganisms, the RuMP pathway has been completely assembled in synthetic methylotrophs and is feasible for the conversion of methanol to metabolites (Antoniewicz, 2019). In *C. glutamicum*, despite the existence of an endogenous metabolic pathway in which the four reactions catalyzed by AdhE, Ald, FdhF, and MshC convert methanol to CO_2_, this strain requires an auxiliary carbon source to maintain cell growth (Witthoff et al., 2013). In order to continuously utilize methanol, the RuMP pathway was introduced to *C. glutamicum* by heterogeneous expression of Mdh from *Bacillus methanolicus* and Hps and Phi from *Bacillus subtilis* that produced an average methanol consumption rate of 1.7 mM/h in a glucose-methanol-containing medium (Witthoff et al., 2015). Simultaneously, a non-methylotrophic strain was successfully modulated to produce ^13^C-label cadaverine from methanol, though high amounts of methanol added to the fermentation medium were toxic to *C. glutamicum* cells (Lessmeier et al., 2015). Accordingly, to enhance the methanol tolerance of *C. glutamicum*, adaptive laboratory evolution was applied to screen mutant strains that exhibited tolerance to high methanol content (Lessmeier and Wendisch, 2015; Wang et al., 2020). Comparative genome analysis of these mutant strains indicated that the amino acid substitution of A165T in MetY and a shortened Cat are crucial factors for improving methanol tolerance (Lessmeier and Wendisch, 2015). In addition, adaptive laboratory evolution was also applied to screen mutant strains capable of increased methanol-dependent cell growth in methanol-xylose-containing medium (Tuyishime et al., 2018). In these mutant strains, the carbon atom in methanol was used to synthesize cell materials, co-factors, and intermediates, which were subsequently converted to L-glutamate through interference with the biosynthesis of the cell wall (Tuyishime et al., 2018). Results from transcriptome analysis illustrate that increased methanol concentrations readjusted the metabolic flux distribution of the glycolysis pathway, amino acid biosynthesis pathway, oxidative phosphorylation reactions, ribosome biosynthesis, and parts of the tricarboxylic acid cycle in *C. glutamicum* (Wang et al., 2020). Moreover, mechanistic analysis of mutant strains obtained by adaptive laboratory evolution of methanol-glucose co-utilizing strains demonstrated that improved expression of *mdh*-*hxlAB*, improved supplementation of riboflavin, and S288N mutation in MetY contribute to the distinct methanol-dependent growth of *C. glutamicum* (Hennig et al., 2020). Inspired by research on methanol bioconversion, a similar study claimed that methyl acetate can be easily obtained from the carbonylation of methanol and CO and that this was utilized by *C. glutamicum* through the introduction of a highly active esterase (Choo et al., 2016). In practice, current progress consistently indicated that cell growth of *C. glutamicum* was hindered by the cytotoxicity of methanol and formaldehyde, which restricted the conversion from methanol to chemicals. In addition to using engineered *C. glutamicum* to improve utilization efficiency and tolerance of methanol, the range of synthetic chemicals produced should also be further expanded.

### Glycerol

Glycerol, a carbon-containing waste resulting from the biodiesel production process, has been widely applied in microbial fermentation owing to its high-reduction property, which frequently leads to higher maximum theoretical yields for producing chemicals (Xiberras et al., 2019). Recently, global markets witnessed remarkable growth in the biodiesel industry that subsequently stimulated research toward utilization of byproduct glycerol (Vivek et al., 2017). Due to its abundance, high-reduction ability, non-toxicity, and low cost, glycerol is regarded as a favorable carbon substrate for numerous industrial microbes, including *Escherichia coli* (Westbrook et al., 2019), *Saccharomyces cerevisiae* (Xiberras et al., 2019), *B. subtilis* (Fan et al., 2018), and *Pseudomonas* species (Pobletecastro et al., 2020) to produce various types of metabolites. Glycerol is typically not treated as a carbon resource for *C. glutamicum*-based bioprocesses due to the absence of glycerol oxidation pathway enzymes in this strain. In practice, *glpK-*encoded glycerol kinase and *glpD-*encoded glycerol-3-phosphate dehydrogenase from *E. coli* are introduced into *C. glutamicum* to obtain engineered strains capable of metabolizing glycerol (Rittmann et al., 2008). In order to further improve glycerol utilization, heterogeneous expression of *glpF*, which encodes a glycerol facilitator from *E. coli*, was developed, accelerating the growth of *C. glutamicum* on glycerol. These strategies have been intensively applied to developing engineered *C. glutamicum* to convert glycerol into chemicals. For instance, overexpression of *E. coli*_*glpFKD* in corresponding *C. glutamicum* strains produced 3.5 ± 0.8 mM, 23.0 ± 2.3 mM, 17.9 ± 0.4 mM, and 23.6 ± 0.9 mM of L-glutamate, L-lysine, L-ornithine, and L-arginine, respectively (Rittmann et al., 2008; Meiswinkel et al., 2013b). Additionally, plasmid-based overexpression of *E. coli*_*glpFKD* in engineered *C. glutamicum* produced 11 ± 1 mM L-pipecolic acid at a yield of 0.14 ± 0.02 g/g glycerol (Pérez-García et al., 2017), 0.6 ± 0.0 g/L ectoine at a yield of 0.055 ± 0.003 g/g glycerol (Perez-Garcia et al., 2017), and 38.4 g/L succinate at a yield of 1.02 g/g glycerol (Wang et al., 2016), indicating that the majority of chemicals can be produced from glycerol in manipulated strains. Although rapid progress has been made in modulation of *C. glutamicum* to convert glycerol to chemicals, there are still uncertainties: At first, crude glycerol obtained from biodiesel production bioprocesses contains growth inhibitors that limit its application for the majority of industrial microorganisms (Vivek et al., 2017). Second, poor metabolic flux to the pentose phosphate pathway, using glycerol as carbon resource, results in inadequate cofactor NADPH regeneration, hindering the biosynthesis of chemicals including amino acids, fatty acids, and others, which requires NADPH to drive multistep enzyme reactions (Xiberras et al., 2019). In conclusion, if these obstacles can be addressed, microbial fermentation will be a promising approach for converting glycerol to metabolites.

### Arabinose

Arabinose is the second most abundant pentose, following xylose, in plant cellulose biomass, and is intensively used as a carbon feedstock for industrial microbes. Assimilation of arabinose requires three enzyme actions and a membrane protein. First, arabinose isomerase (encoded by *araA*) catalyzes L-arabinose to synthesize L-ribulose. Second, ribulokinase (encoded by *araB*) catalyzes L-ribulose to synthesize L-ribulose-5-P. Third, ribulose-5-phosphate 4-epimerase (encoded by *araD*) catalyzes L-ribulose-5-P to synthesize D-xylulose-5-P, and this terminal metabolite is able to enter the pentose phosphate pathway (Gopinath et al., 2012; Zhao et al., 2018; Choi et al., 2019). AraE, a membrane channel protein that functions as a xylose transporter, was able to simultaneously transport L-arabinose. *C. glutamicum* strains, with the exception of *C. glutamicum* ATCC31831, cannot utilize arabinose due to lack of this metabolic pathway (Zhao et al., 2018; Choi et al., 2019). By heterogeneously assembling the arabinose metabolic pathway from *E. coli*, researchers engineered *C. glutamicum* to produce succinic acid from arabinose (Kawaguchi et al., 2008). Subsequently, *C. glutamicum* ATCC31831 with the ability to grow on L-arabinose was discovered by random screening. Analysis of the genome of this strain suggested that genes associated with arabinose utilization were included in the *araBAD* operon, which is negatively controlled by the transcription factor AraR (Kuge et al., 2015). In addition, simultaneous utilization of L-arabinose and D-glucose in *C. glutamicum* ATCC31831 indicated that carbon metabolism repression was ineffective against arabinose in this strain (Kawaguchi et al., 2009). Hence, development of recombinant *C. glutamicum* strains for converting arabinose to chemicals has received extensive attention. Heterologous expression of the *E. coli araBAD* operon in *C. glutamicum* HalT1 enabled fermentation production of 7-Cl-Trp and Trp at a titer of 52 ± 1 mg/L and 2.4 ± 0.1 g/L, respectively (Veldmann et al., 2019). Co-expression of *the E. coli araBAD* operon and *X. campestris xylA* endowed engineered *C. glutamicum* with the ability to simultaneously utilize arabinose and xylose as well as improve the production titers of L-lysine, L-glutamate, L-ornithine, and putrescine (Meiswinkel et al., 2013a). Despite the existence of endogenous arabinose metabolic pathways in *C. glutamicum* ATCC31831, researchers prefer heterologous expression of the *araBAD* operon from *E. coli* to construct engineered *C. glutamicum* strains (Meiswinkel et al., 2013a; Pérez-García et al., 2017). Because hydrolyzed cellulose is composed of glucose, xylose, arabinose, and other sugars, it can be acknowledged that the arduous feasibility of mixed sugar fermentation is the main bottleneck for the utilization of arabinose (Jojima et al., 2015; Mindt et al., 2019b).

### Mannose or Mannitol

In addition to xylose and arabinose, mannose is another feedstock, making up approximately 20% of the sugar composition in lignocellulose hydrolyzate. Engineering recombinant strains to convert mannose to valuable chemicals has captured widespread attention. Mannose catabolism relies largely on the branching metabolism of the glycolytic pathway, similar to other sugars. In this metabolic pathway, mannose is transported into the cytoplasm, accompanied by acquisition of the phosphoryl group from phosphoenolpyruvate to generate mannose-6-phosphate. Subsequently, phosphomannose isomerase is employed to catalyze mannose-6-phosphate to synthesize fructose-6-phosphate, which is an intermediate in the glycolytic pathway. In *C. glutamicum*, a well-established native mannose metabolic pathway consists of glucose or fructose permeases (encoded by *ptsG* or *ptsF*), as well as phosphomannose isomerase (encoded by *manA*), which has been applied for the biosynthesis of various organic acids from mannose (Sasaki et al., 2011). Under anaerobic conditions, co-overexpression of *manA* and *ptsF* not only accelerated utilization of mannose but also broke down carbon metabolite repression, enabling simultaneous metabolism of glucose and mannose in *C. glutamicum* (Sasaki et al., 2011). Currently, engineered *C. glutamicum* to utilize mannose requires deforestation to prepare raw materials that are unsustainable; therefore, attention has been transferred to its reductive format, mannitol, which can be easily obtained from hydrolysis of marine plants.

Similar to mannose, the catabolic pathway of mannitol involves two transmembrane transport systems and two enzymatic reactions; mannitol can also be processed in *C. glutamicum* (Figure 1). First, it is transported from extracellular to intracellular space using the transmembrane protein MtlT and converted to fructose by mannitol dehydrogenase (encoded by *mtlD*) (Peng et al., 2011). Subsequently, fructose is transported twice across the cell membrane to produce fructose-1-phosphate by employing a non-specific PTS transport system composed of an EI (encoded by *ptsIH*) and an EII (encoded by *ptsF*) membrane protein. Moreover, fructose-1-phosphate is applied to generate fructose-1,6-bisphosphate under the catalysis of phosphofructokinase (encoded by *pfkB*), which enters the glycolysis pathway (Laslo et al., 2012). Therefore, it is reasonable to speculate that sufficient expression of mannitol transporter and mannitol dehydrogenase is a critical factor for the utilization of mannitol in *C. glutamicum*. In general, *C. glutamicum* is unable to utilize mannitol until the negative regulator MtlR is removed; this regulator severely represses the expression of *mtlTD* operon (Peng et al., 2011). Accordingly, deletion of MtlR was performed in the lysine-producing strain *C. glutamicum* Lys12 to generate the recombinant strain *C. glutamicum* SEA-1, which is capable of producing 8.5 mM L-lysine from mannitol (Hoffmann et al., 2018). However, the recombinant *C. glutamicum* SEA-1 strain could only metabolically convert mannitol as long as the cell growth rate and biosynthesis of intracellular cofactor NADPH were much lower than in the parent *C. glutamicum* Lys12 strain cultured on glucose (Hoffmann et al., 2018). Fructose was found in the fermentation broth of strain *C. glutamicum* SEA-1, along with secondary growth observed in the fermentation process, indicating that fructose was incompletely converted to fructose-1-phosphate and secreted into the extracellular space. Thus, there are important factors, such as low expression of related enzymes, inefficient fructose uptake system, and insufficient supply of cofactor NADPH, that restrict biotransformation from mannitol to chemicals in MtlR-stripped *C. glutamicum*. Further regulatory mechanism analyses of mannitol metabolism, rational design and modification of related gene targets, and adoption of appropriate metabolic engineering approaches to optimize the metabolic flux of mannitol catabolism and fructose metabolism pathways are crucial for improving mannitol utilization efficiency.

### N-Acetylglucosamine

N-Acetylglucosamine is an amino sugar that serves as a monomer for chitin, a polymer widely present in the exoskeleton of crustaceans. Cooked shrimp serves as food for humans, particularly in China, producing several million tons of shellfish waste, of which chitin makes up approximately half of the dry weight. Simple disposal of these wastes not only wastes biological resources but also causes environmental pollution, detracting from sustainable development of the national economy. Engineering microbes to utilize abundant polysaccharides from crustacean exoskeletons in order to produce chemicals has attracted extensive attention from researchers. Generally, the metabolic pathway of N-acetylglucosamine consists of a specific transport system and two enzyme actions, catalyzed by N-acetylglucosamine-6-phosphatedeacetylase (encoded by *nagA*) and glucosamine-6P deaminase (encoded by *nagB*), converting N-acetylglucosamine to fructose-6-phosphate that enters the glycolytic pathway (Matano et al., 2016). In *C. glutamicum*, the absence of a specific uptake system prevents the utilization of extracellular N-acetylglucosamine, requiring heterogeneous expression of *nagE* from *Corynebacterium glycinophilum*; this expression enables prompt transportation and feasible assimilation of N-acetylglucosamine. Co-expression of exogenous *nagA*, *nagB*, and *nagE* generated *C. glutamicum* capable of producing various chemicals, including L-lysine (Sgobba et al., 2018), L-citrulline (Eberhardt et al., 2014), lycopene (Matano et al., 2014), putrescine (Uhde et al., 2013), 7-chloro-L-tryptophan (Veldmann et al., 2019), 5-aminovalerate (Jorge et al., 2017b), gamma-aminobutyric acid (Jorge et al., 2017a), ectoine (Perez-Garcia et al., 2017), and L-pipecolic acid (Pérez-García et al., 2017) from N-acetylglucosamine. Consequently, N-acetylglucosamine is a promising alternative carbon source for *C. glutamicum*, although further investigation is required to accelerate the conversion from N-acetylglucosamine to bulk chemicals at the industrial scale. Additionally, the solid nature of chitin is unfavorable in preprocessing, as it hinders industrial application of amino sugars.

### Cellobiose

Cellobiose, a disaccharide formed by the connection of two glucose molecules, is a carbon feedstock generated by degradation of cellulose through a synergistic reaction between endoglucanase and cellobiohydrolase (Li et al., 2020). Currently, direct utilization of cellobiose requires a β-glucosidase that catalyzes breakage of β-1,4-glucoside bonds and converts cellobiose to glucose. Due to the absence of a cellobiose transporter and low permeability of cellobiose, expression of β-glucosidase in microorganisms invariably requires secretion or surface display in order for the enzyme to contact the substrate. Traditionally, secreted expression or surface display of β-glucosidase has been applied for biorefinery chemical production in a multitude of industrial strains, including *Clostridium thermocellum* (Dash et al., 2019), *S. cerevisiae* (Yun et al., 2018), *Pseudomonas putida* (Dvoøák and de Lorenzo, 2018), *E. coli* (Satowa et al., 2020), and *C. glutamicum* (Adachi et al., 2013). *C. glutamicum* displays high β-glucosidase activity derived from *Saccharophagus degradans*, successfully displaying β-glucosidase fused with the C-terminus of the anchor protein PorC. On the surface, *C. glutamicum* enables simultaneous saccharification and fermentation, producing three-fold more L-lysine than a β-glucosidase-secretory *C. glutamicum* strain (Adachi et al., 2013). Co-expression of endoglucanase and β-glucosidase, either secretory or surface-displaying, in *C. glutamicum* DM1729 directly converts cellulose to L-lysine (Anusree et al., 2016). However, due to insufficient β-glucosidase activity, the yield of L-lysine and the cellobiose consumption rate of this engineered *C. glutamicum* were underdeveloped. To address limitations on enzyme activity, Tfu0937 from *Thermobifida fusca*, which shares high β-glucosidase activity with *E. coli*, was codon-optimized and introduced into *C. glutamicum*. Fusion with the CgR0949 signal sequence resulted in a Tfu0937-secreting strain that produced 9.7 g/L lysine, an improvement of approximately eight-fold compared to the original β-glucosidase-displaying strains (1.08 g/L) (Matsuura et al., 2019). Additionally, intracellular utilization of cellobiose in *C. glutamicum* is feasible through heterologous expression of codon-optimized *cdt-1*, which encodes a cellobiose transporter, and the *gh1-1* gene from *Neurospora crassa* (Lee et al., 2016). By adaptive evolution, this mutant strain was also applied for the co-utilization of cellobiose and xylose.

## Direct Bioconversion From Plant Biomass Hydrolyzates to Chemicals

Lignocellulosic biomass is generally regarded as agricultural waste, stored in huge amounts of organic carbon sources. Although many cellulosic ethanol factories are gradually coming into operation, lignocellulosic biomass is still underutilized and poses major environmental problems in some economically disadvantaged areas. Thus, there is an urgent need to improve the utilization of lignocellulosic biomass, which is a promising alternative raw material for the biobased production of chemicals. Pretreated by high-temperature acid hydrolysis, lignocellulosic materials such as corn stalk, rice straw, cassava bagasse, and wheat bran can be converted into corresponding hydrolyzates containing various monosaccharides (glucose, xylose, arabinose, etc.) that can be used to provide nutrition for microbes.

### Corn Straw Hydrolyzate

Corn is an economic and food crop that is widely planted in numerous countries. Billions of tons of corn straw biomass are generated annually by harvesting corn. Most corn straw is incinerated, and only a fraction is applied for feeding animals, causing an extreme waste of resources and environmental pollution. To address these problems, corn straw has been preprocessed by mixing with dilute sulfuric acid and mild treatment at high temperature to generate exploitable corn straw hydrolyzate, which contains multitudinous carbohydrates, including glucose, xylose, arabinose, and mannose, which are favorable substrates for microbial fermentation (Choi et al., 2019). However, hydrolyzate generated from dilute-acid pretreatment process invariably contains fermentation inhibitors, including furfural, 5-hydroxymethylfurfural (HMF), and phenolic aldehydes, that affect the growth of microbes such as *C. glutamicum*. Hence, improving the tolerance of *C. glutamicum* to these inhibitors was investigated by adaptive evolutionary analysis to obtain a mutant strain capable of rapid growth on corn straw hydrolyzate (Wang et al., 2018). Exploration of tolerance mechanisms using a transcriptome analysis approach found that overexpression of *CGS9114_RS01115*, which encodes an alcohol dehydrogenase, as well as numerous oxidoreductase genes, accelerated conversion of these aldehyde inhibitors in *C. glutamicum* (Zhou et al., 2019). Meanwhile, excess biotin, which is unavoidable in lignocellulosic hydrolyzate produced by the pretreatment process, results in a rigid cell membrane that hampers secretion of L-glutamate, restricting cellulosic L-glutamate production (Wen et al., 2018). This unfavorable phenomenon can be reversed by extrinsic addition of osmotic agents, such as Tween 40, ethambutol, and penicillin, a step that not only improves the yield of L-glutamate but also promotes accumulation of L-arginine and L-ornithine during fermentation (Chen M. et al., 2015; Jiang et al., 2020). In addition, the uptake system of biotin is reinforced by overexpression of the *bioYMN* operon, which encodes biotin transporter, reducing biotin content in corn straw hydrolyzate and stimulating faster cell growth and multiplicative cellulosic L-glutamate production (Han et al., 2019). Moreover, to increase the transport of L-glutamate, MscCG, the primary L-glutamate transporter, was truncated, which stimulated L-glutamate production in biotin-rich corn stover hydrolyzates (Wen and Bao, 2019; Kawasaki and Martinac, 2020). Reducing biotin concentration in corn stover hydrolyzates is indispensable for cellulosic L-glutamate production. In addition to L-glutamate, L-lysine is a bulk amino acid that is in tremendous global demand and can be produced from corn straw hydrolyzate. For instance, fermentation cultivation of *C. glutamicum* SIIM B253 in high-glucose corn straw hydrolyzate resulted in L-lysine accumulation at a titer of 7.4 g/L. By modulating nutrient concentration, fermentation conditions, and simultaneous saccharification and fermentation processes, the yield of L-lysine was further elevated to 33.8 g/L, representing the highest yield of cellulosic L-lysine (Chen et al., 2019). As illustrated in these examples, *C. glutamicum*-based cellulosic biomass utilization has focused on the assimilation of glucose in corn straw hydrolyzate. Xylose serves as the second most abundant carbon feedstock in corn straw hydrolyzate, though *C. glutamicum* does not contain its own xylose metabolic machinery. Heterologous assimilation of xylose metabolic pathways from *X. campestris* were processed in strain SAZ3 that then produced 98.6 g/L of succinate by two-stage fermentations of glucose and xylose in corn straw hydrolyzate (Mao et al., 2018). However, despite extensive investigations aims at engineering *C. glutamicum* strains to produce valuable chemicals from corn straw hydrolyzate, multitude of problems, including the complexity of nutrients, generation of fermentation inhibitors, complex pretreatment process, and limited sugar yield, still restrict the large-scale application of corn straw hydrolyzate. In summary, corn straw hydrolyzate is an alternative renewable feedstock with huge potential in the field of microbial fermentation if those drawbacks could be addressed.

### Other Cellulose Hydrolyzates

There are many food crops grown on Earth. In addition to corn straw, cellulose hydrolyzate prepared from crop residues such as cassava bagasse, straw stalk, and wheat bran are also applied for the cultivation of industrial microbes. Among these, cassava bagasse is a residuum generated from the starch extraction process and frequently identified as widespread waste, occupying a high proportion of disposal capacity (Padi and Chimphango, 2020). Engineering microbes for bioconversion of cassava bagasse has aroused intense interest in the past few decades (Sugumaran et al., 2014). Currently, the enzymatic hydrolysis products of cassava bagasse contains amounts of glucose and trace amounts of xylose, arabinose, and acids has been resoundingly used for the bioproduction of various bulk chemicals, including alcohols (Huang et al., 2019), organic acids (Wang and Yang, 2013), and fatty acids (Chen J. et al., 2015). For example, an immobilized *C. glutamicum* cell device, using a mixture of substrates and alkalis, has been discovered and can produce succinic acid from cassava bagasse (Shi et al., 2014). After immobilization in a porous polyurethane filler, *C. glutamicum* can be used for cyclic utilization of hydrolyzate generated by the two-step enzymatic hydrolysis of cassava bagasse, producing 22.5 g/L succinic acid for each round of fermentation (Shi et al., 2014). Additionally, enzymatic hydrolysis of cassava bagasse (contains (w/w) 50.3% starch, and 12.2% fiber, and 6.5% moisture) recovers approximately half the mass of glucose (0.53 g glucose/g cassava bagasse), which can then be utilized by engineered *C. glutamicum*. This produced 18.5 g/L of 5-aminolevulinic acid during fed-batch fermentation, exhibiting 90.1% thrift on the cost of carbon material (Chen et al., 2020). In addition to cassava, rice is another widely planted food crop that generates a hundred billion tons of waste straw in the harvested phase. Traditionally, incineration disposal of rice straw has resulted in extensive haze and waste of resources. Acid treatment of rice straw generates 42 g/L of carbohydrate, containing 40 mM glucose, 166 mM xylose, and 66 mM arabinose, which can be utilized by engineered *C. glutamicum* containing a heterogeneously assembled xylose and arabinose metabolic pathway and produced 96 mM of L-glutamate during 100 h of fermentation cultivation (Gopinath et al., 2011). Similarly, acid treatment of wheat bran generates 41 g/L of carbohydrate, containing 125 mM glucose, 62 mM xylose, and 64 mM arabinose, was also can be utilized by engineered *C. glutamicum* to produce L-glutamate (Gopinath et al., 2011). Inspired by this example, biobased production of *N*-ethylglycine from rice straw hydrolyzate was enabled in engineered *C. glutamicum* by introducing a mutant DpkA from *P. putida* (Mindt et al., 2019a). Moreover, small quantities of plant biomass hydrolyzates, such as those derived from sorghum, softwood lignin, and *Miscanthus*, also deserve attention in the fermentation field. Biobased production of 5-aminovaleric acid (Joo et al., 2017) and biogasoline isopentenol (Sasaki et al., 2019) from *Miscanthus* hydrolyzate, as well as *cis-*muconic acid from depolymerized small aromatics of softwood lignin (Becker et al., 2018a), is feasible. Biomass hydrolyzates provide an alternative to glucose, creating enormous potential to alleviate the problem of industrial fermentation competing with humans for food.

## Conclusion and Perspectives

In this review, non-food carbon sources for the fermentation cultivation of *C. glutamicum* and chemical production were summarized. These feedstocks, including xylose, methanol, arabinose, glycerol, mannitol, N-acetylglucosamine, cellobiose, and cellulose hydrolyzates, provide alternative and renewable substrates to produce biobased chemicals. Microbial fermentation using these substrates is expected to alleviate the problem of food competition between industrial fermentation and human nutrition. However, there are still many technical bottlenecks in the practical application of these carbon sources. First, the tolerance of *C. glutamicum* to toxic materials, such as methanol and cellulose hydrolyzate, requires additional improvement to meet the demands of industrial fermentation. Fermentation inhibitors, including furfurals and phenolic aldehydes generated in the dilute-acid hydrolysis process of cellulose, are crucial factors restricting cellulose conversion. In addition to fermentation inhibitors, some nutrients may themselves hinder biosynthesis and secretion of target chemicals. Second, the instability of allogenically assembled metabolic pathways frequently restricts the biosynthesis of chemicals. These metabolic pathways disrupt the original metabolic balance, resulting in low growth and relatively low substrate utilization rates. Dynamic regulation of allochthonous metabolic pathways and chromosome insertion of genes involved in substrate utilization provide an effective solution for unstable pathway engineering. Third, the abundance and outlook of the alternative feedstock are important factors to consider when evaluating the technological index. As sea levels rise, land desertifies, the total human population of the earth increases, and the area of arable land decreases; biomass from terrestrial plants will no longer be the ideal substrate source. Utilization of marine biomass in the form of mannitol, mannose, and trehalose is one alternative, owing to the heavy specific proportion of these substrates in oceans. Meanwhile, stimulated by the development of sea-rice (Chen R. et al., 2017) and sand rice (Genievskaya et al., 2017), the utilization of rice straw hydrolyzate will also play an important role in industrial fermentation in the future.

## Author Contributions

All authors listed have made a substantial, direct and intellectual contribution to the work, and approved it for publication. BZ wrote and submitted this manuscript. YJ and ZL revised the “Bioconversion of Single-Carbon Sources to Valuable Chemicals” section. FW and X-YW revised the “Direct Bioconversion from Plant Biomass Hydrolysates to Chemicals” section.

## Conflict of Interest

The authors declare that the research was conducted in the absence of any commercial or financial relationships that could be construed as a potential conflict of interest.
